# Development of an Independent Prognostic Signature Based on Three Hypoxia-Related Genes for Breast Cancer

**DOI:** 10.1155/2022/2974126

**Published:** 2022-11-03

**Authors:** Hui Wang, Yu Guo, Peipei Zhang, Zhijun Lin, Di Yang, Jiaohong Chen, Zhanzhan Li, Chi Zhang, Haoyu Yang, Binghui Yan, Zhimin Han, Chuntao Tian

**Affiliations:** ^1^Department of Thyroid and Breast Surgery, Sanmenxia Central Hospital of Henan Province, 472000 Sanmenxia, Henan, China; ^2^Personnel section, Sanmenxia Central Hospital of Henan Province, 472000 Sanmenxia, Henan, China; ^3^Department of Radiotherapy, Sanmenxia Central Hospital of Henan Province, 472000 Sanmenxia, Henan, China; ^4^Department of Medical Image Center, Sanmenxia Central Hospital of Henan Province, 472000 Sanmenxia, Henan, China; ^5^Department of Pathology, Sanmenxia Central Hospital of Henan Province, 472000 Sanmenxia, Henan, China; ^6^Community Service Center, Sanmenxia Central Hospital of Henan Province, 472000 Sanmenxia, Henan, China; ^7^Department of Ultrasonography, Sanmenxia Central Hospital of Henan Province, 472000 Sanmenxia, Henan, China; ^8^Department of Oncology, Sanmenxia Central Hospital of Henan Province, 472000 Sanmenxia, Henan, China

## Abstract

**Background:**

Hypoxia was considered to be a prognostic indicator in a variety of solid tumors. This study aims at identifying the hypoxia-related genes (HRGs) in breast cancer (BC) and the feasibility of HRGs as a prognostic indicator.

**Methods:**

We downloaded the mRNA expression data of BC patients from TCGA and GEO databases. The LASSO Cox regression analysis was applied to screen the hub HRGs to establish a prognostic Risk Score. The independence of Risk Score was assessed by multivariate Cox regression analysis. And the immune checkpoint analysis was also performed. In addition, we also detected the expression level of hub HRGs in MCF-10A cells, MCF-7 cells, and SK-BR-3 cells by RT-qPCR.

**Results:**

Three HRGs were identified as hub genes with prognostic value in BC, including CA9, PGK1, and SDC1. The Risk Score constructed by these three genes could efficiently distinguish the prognosis of different BC patients and has been shown to be an independent prognostic indicator. In the high-risk group, patients had lower overall survival and poorer prognosis. In addition, the expression levels of five immune checkpoints (PD1, CTLA4, TIGIT, LAG3, and TIM3) in the high-risk group were significantly higher than those in the low-risk group. Moreover, the expression levels of PGK1 and SDC1 in BC cells were significantly increased.

**Conclusion:**

In this study, we established an efficiently model based on three optimal HRGs (CA9, PGK1, and SDC1) could clearly distinguish the prognosis of different BC patients.

## 1. Introduction

Breast cancer (BC) is one of most common malignancy in women, resulting in a severe decline in women's quality of life [1]. Among the malignant diseases, BC accounts for 23% of all cancer deaths, seriously threatening women's health [2]. Modern treatment for BC is multimodal, including surgery, radiation, and drug therapy; moreover, it has also been demonstrated that patients with early BC, locally advanced disease, and locoregional relapse could be cured [3]. Despite of advances in diagnosis and treatment of BC, approximately 12% of BC patients eventually developed tumor metastatic, and the 5-year survival rate was only 26% [4]. Therefore, identification of effective prognostic biomarkers contributes to developing personalized therapy and extending the scope of treatment for BC.

Tissue hypoxia was one of the pathological characteristics of malignant solid tumors, leading to tumor progression and refractory treatment [5]. Moreover, hypoxia could directly (through inhibiting T cell proliferation and producing effector cytokines) or indirectly (by metabolic competition, upregulating coinhibitory receptors, or recruiting/transforming immunosuppressed cell populations) induce immunosuppression [6, 7]. In human cancers, tumor hypoxia was considered to be an indicator of poor prognosis, which could reduce the efficacy of surgical resection, radiotherapy, and chemotherapy [8, 9]. Recently, hypoxia-related genes (HRGs) have been considered as valuable biomarkers for the prognosis or curative effect in tumors. For instance, Yang et al. established a HRG signature with strong prognostic value for patients with prostate cancer [10]. Dao et al. identified and validated a reliable hypoxia-related survival score in IDH-mutant glioma stem cells based on five HRGs (LYVE1, FAM162A, WNT6, OTP, and PLOD), which was significantly related to the survival of patients with glioma [11]. Cai et al. constructed and validated a prognostic model for hepatocellular carcinoma (HCC) composed of three hypoxia genes (ENO1, UGP2, and TPI1), which was shown to be effective for the prognosis of HCC patients [12]. Although previous study indicated that downregulated hypoxia transcriptome *in vitro* was closely related to the depressed prognosis in BC [13], the prognostic values of HRGs in BC was still unclear and attract us to further study on it.

In the present study, we established a Risk Score for BC patients' prognosis based on three optimal HRGs (CA9, PGK1, and SDC1). Moreover, this predictive model could predict the prognosis of BC patients and should provide novel clues for prognostic stratification.

## 2. Materials and Methods

### 2.1. Data Collection

The clinical information and mRNA profile data of 1092 BC patients were obtained from TCGA database (https://tcga-data.nci.nih.gov/tcga/). We eliminated 10 inappropriate samples, and the remaining 1082 samples were randomly divided into two groups: training set (*N* = 541) and testing set (*N* = 541). The clinical information of BC patients in the two groups was provided in Table 1. In addition, we also obtained two mRNA expression profiles (GSE42568 and GSE48391) and corresponding clinical information from the GEO database (https://www.ncbi.nlm.nih.gov/geo/), which were combined as the verification set to determine the accuracy of the predictive model. These two GEO datasets included 186 BC patients totally, and all the data were detected by using the Afymetrix Human Genome U133 Plus 2.0 Array.

### 2.2. Screening of HRGs

In this study, a total of 26 HRGs were taken into consideration. These genes were derived from previous studies, and most of them have been proven to play a key role in the prognosis of a variety of cancers, including esophageal cancer, laryngeal cancer, and HCC [14–16]. The information of the 26 HRGs was provided in Table S1.

### 2.3. Consensus Clustering Analysis

Based on the mRNA expression values of the 26 HRGs, the samples were clustered by the “*ConsensusClusterPlus*” package of the R software [17].

### 2.4. The Establishment of Risk Score Model

Based on the expression values of 26 HRG, BC samples were analyzed by univariate Cox regression, and the genes were screened which significantly associated with the prognosis of BC (*P* < 0.05). The candidate genes associated with prognosis were further screen via LASSO Cox regression analysis, and finally hub genes were obtained [18]. The Risk Score was constructed based on hub HRGs as follows:

(1) $$\begin{array}{c} \text{Risk Score}=\overset{n}{\underset{i=1}{\sum }}{{\text{Coef}}_{i}}^{*}X_{i}. \end{array}$$

In this formula, Coef_*i*_ (risk coefficient of each HRG) was calculated by LASSO Cox regression analysis, and *X*_*i*_ represented the expression level of each HRG. The optimal cutoff value of the Risk Score was determined by the *survival* package and *survminer* package of R software using the bilateral log-rank test. Then, all BC samples were divided into the following two groups based on the optimal cutoff value: the high-risk group and low-risk group.

### 2.5. Survival Analysis

The Kaplan-Meier method was used to evaluate the overall survival (OS) probability of all groups by the *survival* and *survminer* packages of R software, and the subsequent significance was determined via log-rank test. The *survival* ROC package of R software was used to plot the time-dependent receiver operator characteristic (ROC) curve [19].

### 2.6. Proportion of Infiltrating Immune Cells

The CIBERSORT was a widely used method to assess the composition of immune cells in tumor microenvironment [20]. In our study, the CIBERSORT algorithm was used to evaluate the infiltration level of 22 immune cells in each BC sample.

### 2.7. Nomogram Analysis

A nomogram model was constructed by the *RMS* package of R language to predict the survival probability of BC patients for one-, three-, and five-year based four independent prognostic factors (Risk Score, age, radiation therapy, and TNM Stage). The calibration curve of nomogram was plotted to determine the relationship between the actual probability and predicted probability.

### 2.8. Cell Culture

The human mammary epithelial cells (MCF-10A) and BC cell lines (MCF-7 and SK-BR-3) were provided by ATCC (American Type Culture Collection, Manassas, VA, USA). MCF-10A cells were cultured in MEpiCM Medium (ScienCell) supplemented with 10% FBS (Gibco), 1% MEpiCGS (ScienCell), and 1% penicillin/streptomycin (PS, ScienCell) at 37°C in 5% CO_2_. MCF-7 cells were cultured in DMEM (Gibco) supplemented with 10% FBS (Gibco), 0.01 mg/mL human recombinant insulin (HRI, Solarbio), and 1% PS (HyClone) at 37°C in 5% CO_2_. SK-BR-3 cells were cultured in RPMI 1640 (Gibco) supplemented with 10% FBS (Gibco) and 1% PS (HyClone) at 37°C (no CO_2_).

### 2.9. Real-Time Quantitative PCR (RT-qPCR)

Total RNAs of cells were extracted by TRNzol Universal (TIANGEN BIOTECH (BEIJIN) CO., LTD. China). Nanodrop 2000 (Thermo, USA) was used to detect the quantification and concentration of total RNAs. Next, the total RNAs were reversely transcribed into cDNAs with RevertAid First Strand cDNA Synthesis Kit (Thermo, USA) and then used to perform RT-qPCR with TB Green® Premix Ex Taq™ II (Takara, Japan). RT-qPCR thermocycling protocol was as follows: initial denaturation at 95°C for 30 s, denaturation at 95°C for 10 s, annealing at 60°C for 30 s, and amplification for 40 cycles. GAPDH was used as the housekeeping gene. The primer sequences were shown in Table 2. The 2^−△△CT^ method was applied to calculate the expression level of genes and normalized to GAPDH.

### 2.10. Statistical Analysis

We used R software v3.5.2. for statistical analysis. The Mann–Whitney tests were used to analyze the infiltration differences of immune cells among all groups, with *P* < 0.05 was considered statistically significant.

## 3. Results

### 3.1. The Expression of HRGs Was Correlated with the OS of BC Patients

**Figure 1:**
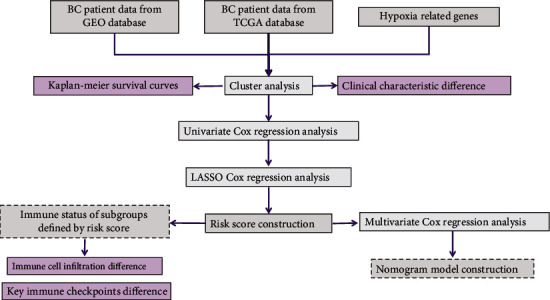
The flow chart of this work.

**Figure 2:**
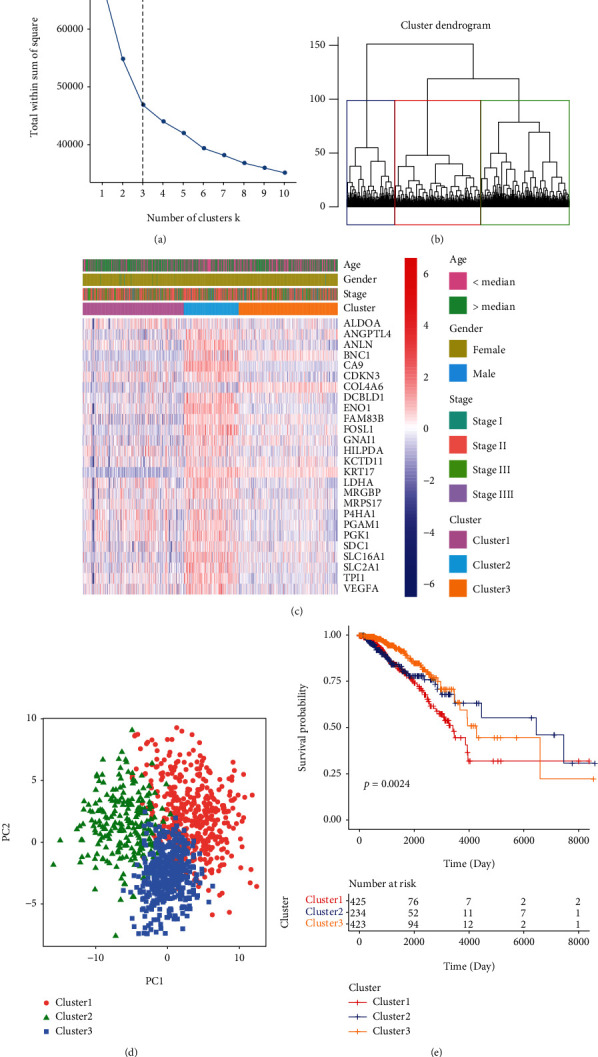
The consensus clustering analysis of BC samples based on the mRNA expression values of 26 HRGs. (a) The elbow chart to determine the optimal number of clustering. The horizontal axis: the numbers of clustering (k) and the vertical axis: the sum of squared error. The point where the decline tending to be flat was the optimal number of clustering (*k* = 3). (b) The consistency matrix of samples and different colors represented different clusters. (c) The heat maps of expression values of HRGs in three clusters. The horizontal axis represented genes; the vertical axis represented samples; red indicated high expression; blue indicated low expression. (d) PCA analysis. The dots with different colors and shapes represented different groups of samples and the closer two dots, the more similar the expression of HRGs in the samples. (e) The Kaplan Meier survival curve of three clusters. *P* value was calculated by log-rank test.

To better display the process building the hypoxia-related prognostic signature of BC, the flow chart of this work was shown in Figure 1. Firstly, K-mean clustering analysis was performed on BC samples according to the expression levels of 26 HRGs, and all BC patients were divided into 3 clusters (*k* = 3) (Figure 2(a)). The results of consensus clustering (Figure 2(b)) and the heat map of expression values (Figure 2(c)) exerted a better clustering effect, suggesting that the three clusters could be efficiently separated. Meanwhile, principle component analysis (PCA) suggested that there were significant differences among the three clusters (Figure 2(d)). Moreover, the Kaplan-Meier curves demonstrated that there were significant differences in OS among three clusters (Figure 2(e)). These results indicated that all the BC patients with different prognosis could be efficiently separated through the expression levels of these 26 HRGs, suggesting the potential predictive values of HRGs in BC prognosis.

### 3.2. The Predictive Model Could Effectively Predict the OS of BC Patients

**Figure 3:**
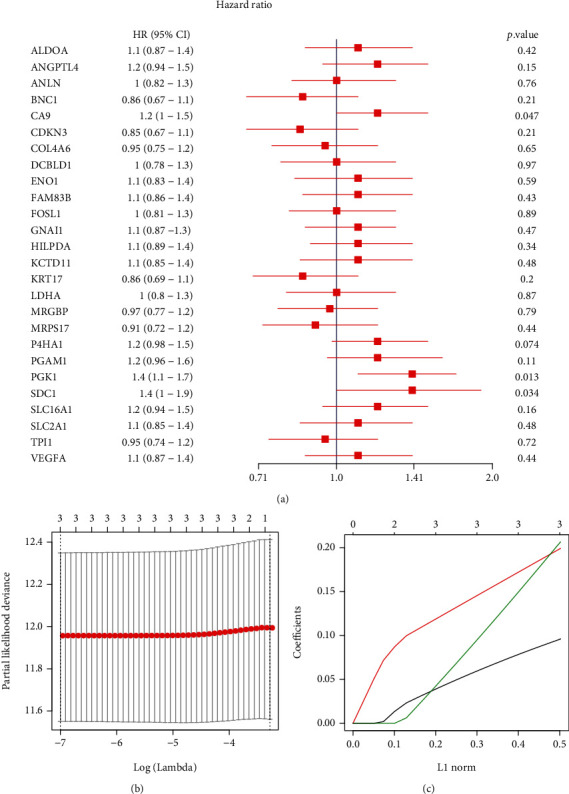
The construction of the prognostic model for BC. (a) The forest plot of Univariate Cox regression analysis with 26 HRGs which were significantly related to the prognosis of BC. HR: hazard ratio; 95% CI: the 95% confidence interval. (b) The tuning parameter lambda was determined by LASSO regression analysis. The optimal lambda value was obtained after taking the log below the dotted line, and the number of variables was corresponding to the top of the optimal lambda. (c) The coefficient spectrum of LASSO Cox regression model.

We next used Univariate Cox regression analysis to calculate the hazard ratio (HR) of 26 HRGs, and the results showed that CA9, PGK1, and SDC1 (*HR* > 1.0, *P* < 0.05), were significantly associated with the OS of BC patients (Figure 3(a)), indicating that these three genes were risk genes, and their high expression was associated with poor prognosis. Further, LASSO Cox regression analysis also showed that these three genes were significantly associated with the prognosis of BC patients (Figures 3(b) and 3(c)).

**Figure 4:**
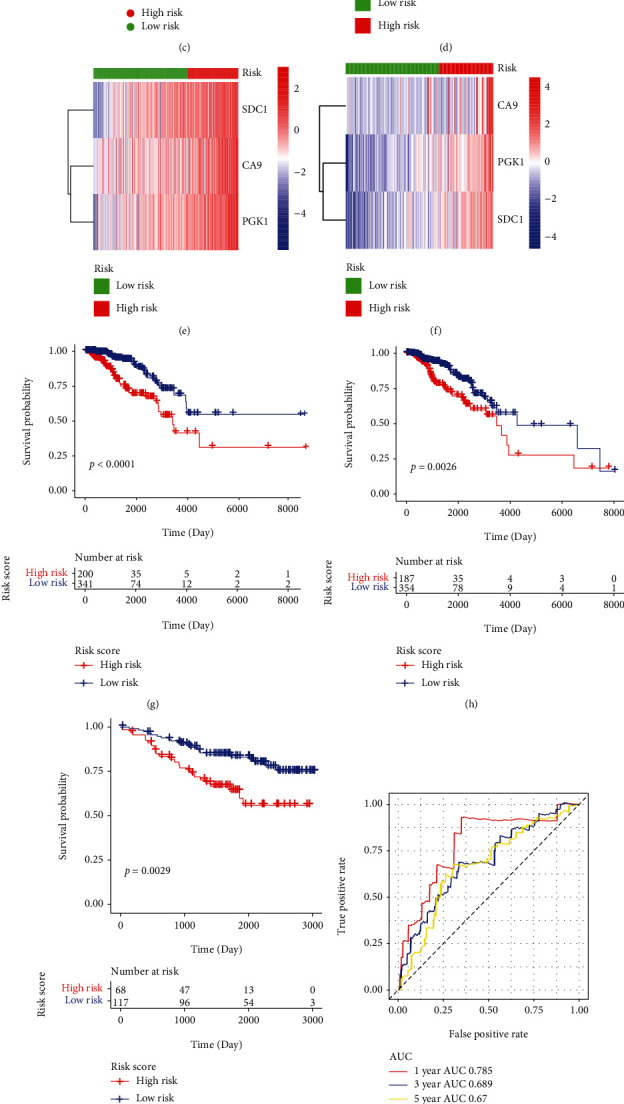
The prognostic model could efficiently predict the survival of BC patients. (a–c) The Risk Score distribution of samples from TCGA database and GEO cohort. One point represented a sample; the red points were the samples with high Risk Score; the green points were the samples with low Risk Score, and the intersection point was the optimal Risk Score. (a) Training set from TCGA database, (b) Testing set from TCGA database, and (c) GSE42568 and GSE48391 cohorts. (d–f) The heat map of mRNA expression values of three HRGs in samples from TCGA database and GEO cohort. The horizontal axis represented genes; the vertical axis represented samples; red indicated high expression; blue indicated low expression. The categories of samples were marked with different colors on the top of the heat map. (d) Training set from TCGA database, (e) Testing set from TCGA database, and (f) GSE42568 and GSE48391 cohorts. (g–i) The Kaplan Meier survival curve of samples from TCGA database and GEO cohort. (g) Training set from TCGA database, (h) Testing set from TCGA database, and (i) GSE42568 and GSE48391 cohorts. (j–l) The time-dependent ROC curve of samples from TCGA database and GEO cohort. The horizontal axis was False Positive; the vertical axis was True Positive; the accuracy of prediction was evaluated by AUC value (the area under the ROC curve). (j) Training set from TCGA database, (k) Testing set from TCGA database, and (l) GSE42568 and GSE48391 cohorts.

Next, these three genes were used to construct a Risk Score model for prognosis of BC patients. First, we calculated the expression levels of these three genes in the TCGA dataset, the GSE42568 cohort, and the GSE48391 cohort; then, we standardized and normalized the expression values. Normalization was Coef_*i*_ weighting of gene expression values using LASSO Cox regression analysis. Subsequently, the formula of Risk Score was obtained as follows: Risk Score = 0.0962^∗^ express value of CA9 + 0.1993, ^∗^ express value of PGK1 + 0.2067, and ^∗^ express value of SDC1. We calculated the Risk Score of each sample and then divided all samples from TCGA database and GEO database into two groups based on the optimal cut-off point (0.1137): low-risk group and high-risk group. The Risk Score distribution of all samples was shown in Figures 4(a)–4(c). As shown in Figures 4(d)–4(f), the expression values of the three genes were significantly different between two groups. Moreover, the results of survival analysis demonstrated that BC samples from high-risk group had a lower OS than that from low-risk group (Figures 4(g)–4(i)). In addition, the results of time-dependent ROC curves indicated that the area under curve (AUC) values of 1-, 3-, 5-year survival of samples from the training set were 0.785, 0.689, and 0.67, respectively; the AUC values of 1-, 3-, 5-year survival of samples from the testing set were 0.595, 0.671, and 0.631, respectively; the AUC values of 1-, 3-, 5-year survival of samples from verification set were 0.628, 0.603, and 0.637, respectively (Figures 4(j)–4(l)), suggesting that the Risk Score could efficiently predict the prognosis of BC patients. In general, the Risk Score constructed by three hypoxia genes could distinguish the prognosis of different BC patients.

We also validated the expression of these three genes in BC and paracancer samples, and the results showed that the expression levels of the genes were higher in tumor samples (Figures S1(a–c)). Among which, the upregulated levels of PGK1 and SDC1 were more significant. Hence, we selected PGK1 and SDC1 with the most significant differences for RT-qPCR verification.

### 3.3. Immune Status of Subgroups Defined by Risk Score

**Figure 5:**
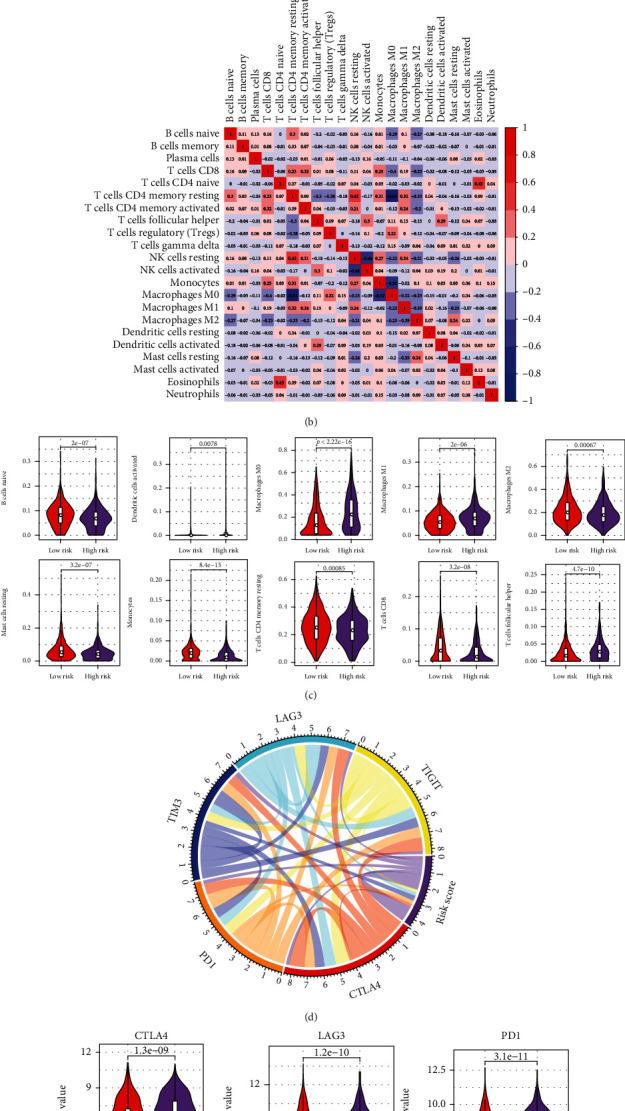
Immune status of BC samples in two subgroups. (a) The relative proportion of immune infiltration in all BC samples. (b) The correlation matrix of 22 immune cell proportions. Red indicated positive correlation; blue indicated negative correlation. The darker the color, the greater the correlation. (c) The violin plot of immune cells with significantly different proportions of infiltration in the high- and low-risk groups. The horizontal axis: high- or low-risk group and the vertical axis: the relative proportion of infiltrating immune cells. *P* value was determined by Wilcoxon test. (d) The correlative circos diagram between Risk Score and the expressions of five prominent immune checkpoints. (e) The violin plot of immune checkpoints with significantly different expressions between high- and low-risk groups.

Hypoxia in solid tumor tissue may be involved in the formation of immunosuppressive microenvironment, resulting in the difficulty of immunotherapy [6, 7]. We next employed CIBERSORT and LM22 eigenmatrix to assess the immune microenvironment composition of the two subgroups defined by Risk Score. The results of immune cell infiltration in all BC samples from TCGA database were shown in Figure 5(a); we also found that there was a weaker correlation in the proportion of infiltration of 22 immune cells (Figure 5(b)), suggesting that the infiltration of different immune cells was more heterogeneous in BC patients. Moreover, there were significant differences in the proportions of ten infiltrating immune cell types, including three types of macrophages (M0, M1, and M2), Monocytes, B cells naive, Dendritic cells activated, Mast cells resting, T cells CD4 memory resting, T cells CD8, and T cells follicular helper between two subgroups (Figure 5(c)). In the high-risk group, the samples had lower proportions of infiltrating B cells naive, Monocytes, Mast cells resting, Macrophages M2, T cells CD4 memory resting, T cells CD8, and higher proportions of infiltrating Dendritic cells activated, Macrophages M0, Macrophages M1, T cells follicular helper, which might account for the prognostic difference in BC patients from these two subgroups.

Recently, the immune checkpoints have emerged as potential biomarkers for cancer immunotherapy [21]. Here, we found that Risk Score was closely correlated with the expression levels of five immune checkpoints, TIGIT, TIM3, PD1, LAG3, and CTLA4 (Figure 5(d)). In addition, compared with the low-risk group, these five immune checkpoints were significantly highly expressed in high-risk group (*P* < 0.05) (Figure 5(e)). It has been known that the immune checkpoints contributed to maintaining an immunosuppressive microenvironment for tumor cells to escape immune surveillance [22]. These results suggested that the poor prognosis of BC patients with high Risk Score might be associated with the immunosuppressive microenvironments.

### 3.4. Risk Score Was Shown to Be an Independent Prognostic Factor

**Figure 6:**
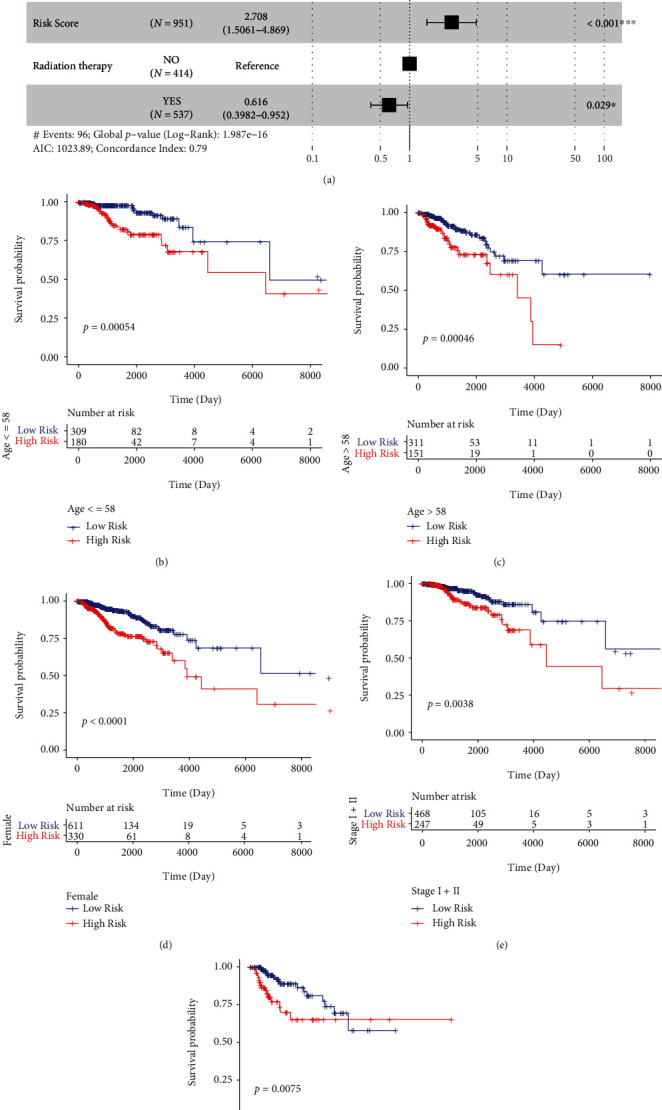
Risk score was an independent prognostic signature for BC. (a) The forest plot of multivariate Cox regression analysis. Compared with the reference samples, the samples with Hazard ratio > 1 had a higher risk of death, while the samples with Hazard ratio < 1 had a lower risk of death. (b–f) The Kaplan Meier survival curves of BC samples with different clinicopathologic factors including (b) age ≤ 58, (c) > 58, (d)female, (e)Stage I + II, and (f) Stage III + IV. *P* value was calculated by log-rank test.

To determine whether Risk Score was an independent prognostic factor, we included Risk Score, gender, age, TNM Stage, and radiation therapy in a multivariate Cox regression analysis. We found that the Risk Score was significantly associated with the OS of BC patients, and the samples with high Risk Score had a higher risk of death (Figure 6(a), HR = 2.708, 95% CI: 1.5061-4.869, *P* < 0.001) compared with those with low Risk Score. Notably, the OS of the samples in the high-risk group was significantly lower than that in the low-risk group (Figures 6(b)–6(f)); survival analysis for male BC patients was not performed since there were only 12 male patients, indicating that the prediction of BC prognosis by Risk Score was not affected by these factors, and Risk Score could be used as an independent prognostic signature to predict the prognosis of BC patients.

### 3.5. The Nomogram Prediction Model

**Figure 7:**
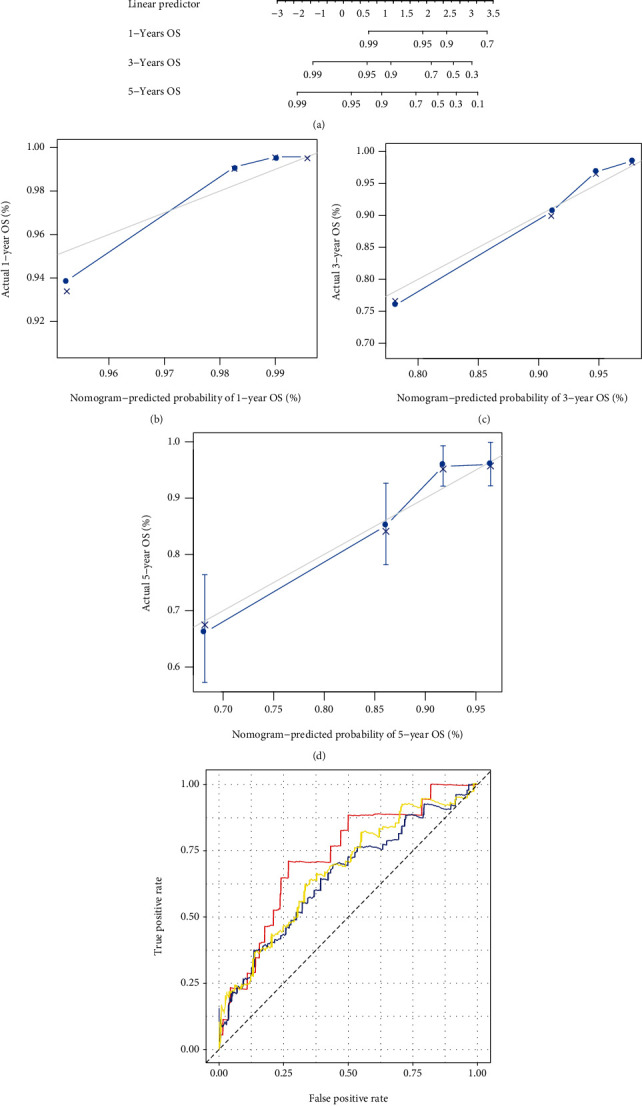
The nomogram model could better predict the long-term survival probability of BC patients. (a) The nomogram for predicting the 1 -, 3 -, and 5 - year OS probability in BC patients. (b–d) The calibration curves of OS probabilities at (b) 1, (c) 3, and (d) 5 years in BC patients. The horizontal axis represented survival rate predicted by nomogram; the vertical axis represented actual survival rate. (e) The time-dependent ROC curves of the nomogram for 1 -, 3 -, and 5 - year OS in BC patients.

Finally, the nomogram model was established based on the four independent prognostic factors including Risk Score, radiation therapy, age, and TNM Stage (Figure 7(a)). The results showed that the corrected curves for 1- (Figure 7(b)), 3- (Figure 7(c)), and 5-year (Figure 7(d)) were closer to the ideal curves (a straight line with a slope of 1 passing through the origin of the coordinate axis), indicating that the prediction was in powerful agreement with the actual results. Meanwhile, the AUC values of nomogram for 1-, 3-, and 5-year were 0.728, 0.651, and 0.673, respectively (Figure 7(e)). These results suggested that the nomogram model could reliably predict the long-term survival probability of BC patients.

### 3.6. Validation of Prognosis-Related Genes by RT-qPCR

**Figure 8:**
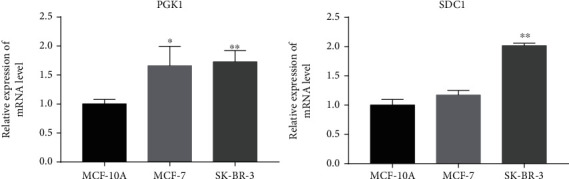
The expression level of PGK1 and SDC1 in BC cells. (Compared with MCF-10A cells, ^∗^*P* < 0.05 and ^∗∗^*P* < 0.01).

Previous studies have shown that the high level of intracellular PGK1 was related to tumorigenesis, progression, and chemoradiotherapy resistance [23]. And the high level of PGK1 was indicative of undesirable overall survival for various cancers [24]. In addition, the high level of SDC1 was also considered to be related to more aggressive tumors and a worse prognosis of prostate cancer [25]. In this study, we found that compared with MCF-10A cells, the expression level of PGK1 was significantly increased both in MCF-7 cells and SK-BR-3 cells (Figure 8), and the expression level of SDC1 was significantly increased in SK-BR-3 cells, while only slightly increased in MCF-7 cells (Figure 8). Such data were consistent with the bioinformatics analysis, indicating that the Risk Score constructed based on HRGs was reliable to evaluate the prognosis of BC patients.

## 4. Discussion

BC has become the most common leading cause of tumor-related mortality among women in the world [26]. Increasing prognostic signatures have been evidenced to show great significance in various tumors [27]. Hence, the identification of efficient prognostic signatures will contribute to the diagnosis and treatment of BC. Recently, HRGs in BC have attracted more attention due to their crucial function closely associated with the development or diagnosis of BC, and even might be the potential therapeutic targets. Duechler et al. revealed that the heterogeneous immune microenvironment in BC was significantly affected by HRGs, and suggested that targeting HRGs might not only sensitize breast tumor for radiation and chemotherapies but also interfere with cancer immunosuppression [28]. Guerrab et al. found that the quantification of HRG expression might be considered as a potential approach for the prediction of clinical outcome in BC [29]. These reports all confirmed the important roles of HRGs in BC. However, the research about the prognostic values of HRGs in BC is lacking. In the present study, we were the first to explore the prognostic values of HRGs in BC and identified three HRGs, including CA9, PGK1, and SDC1, which were closely associated with the prognosis of BC patients, suggesting their potential prognostic values.

The expression of cell-surface carbonic anhydrases IX (CA9) was significantly upregulated in hypoxia for all BC cell lines including MCF7, ZR-75.1, and MDA-mb231 cells and has been demonstrated to be novel therapeutic targets for BC [30, 31]. The mitochondrial translocation of phosphoglycerate kinase 1 (PGK1) was induced by hypoxia [32], and Fu et al. determined that PGK1 was a potential survival biomarker and invasion promoter through modulating the HIF-1*α*-mediated process of epithelial-mesenchymal transition (EMT) in BC [33]. Although the role of SDC1 in BC remains unclear, its crucial function in other human cancer has been studied in detail. Syndecan-1 (SDC1), also known as CD138, can induce an immature and stem cell-like transcriptional program in myeloma cells [34]. In addition, SDC1 has been the gold-standard surface marker to detect multiple myeloma (MM) cells for decades [35]. These studies suggested that the three HRGs play essential functions in various human cancers including BC. Here, a predictive model for prognosis in BC was established based on the three HRGs. Moreover, three datasets composed of training set, testing set, and verification set were all applied to determine the accuracy of this model, which revealed that the prognostic model could efficiently predict the prognosis of BC patients. On the other hand, considering the essential role of hypoxia in various tumors, the pathogenic or therapeutic target potential of CA9, PGK1, and SDC1 in BC should be investigated in our future work.

In the last decades, BC is not generally viewed as a highly immunogenic cancer, but recent studies have described a rich tumor immune microenvironment in BC [36]. Soysal et al. revealed that various components of BC microenvironment, such as suppressive immune cells and altered extracellular matrix, function together to prevent effective antitumor immunity and promote the progression and metastasis of BC [37]. In this study, we analyzed the immune infiltration differences of 22 immune cells in BC samples from high-risk group and low-risk group and found that there were significant differences in the proportions of ten types of infiltrating immune cells in BC patients from high- and low-risk groups. Our analysis was in agreement with previous studies that a rich tumor immunoreaction occurred during the progression of BC, which might account for the prognostic difference in BC patients. Accordingly, our hypoxia-related signature might be helpful to choose appropriate immunotherapy for BC patients, which deserved further exploration in near future.

Although our results suggested that SDC1 might be related to the prognosis in BC, its specific function or mechanism in BC progression should be explored; meanwhile, more samples are needed to be collected to verify the accuracy of our prognostic model.

## 5. Conclusion

In summary, our study established a predictive model based on three HRGs (CA9, PGK1, and SDC1) and demonstrated that this model could reliably predict the prognosis of patients with BC. Our prognostic signature provides an additional alternative for BC prognosis prediction, which will indirectly benefit for better clinical decision and treatment strategies of BC patients.

## Figures and Tables

**Table 1:** The clinical information of samples in the two groups (training set and testing set) from TCGA database.

Characteristics	Groups	Patients samples			*X* ^2^	*P* value
		Total (*N* = 1082)	Training cohort (*N* = 541)	Testing cohort (*N* = 541)		
		Number	Number	Number		
Sex	Female	1070	533	537	1.3483	0.5096
	Male	12	8	4		

Age at diagnosis	Median	58	58	58	0	1
	Range	26-90	26-89	26-90		

Pathological TNM stage	I	182	90	92	1.8478	0.9974
	II	615	304	311		
	III	243	123	120		
	IV	20	12	8		
	X	11	7	4		
	Unknown	11	5	6		

Vital status	Alive	928	470	458	1.0902	0.5798
	Dead	154	71	83		

Radiation therapy	NO	424	222	202	2.1816	0.7024
	YES	547	269	278		
	Unknown	111	50	61		

Disease type	Ductal and lobular neoplasms	1039	518	521	8.3634	0.7561
	Cystic, mucinous and serous neoplasms	16	5	11		
	Complex epithelial neoplasms	14	10	4		
	Adenomas and adenocarcinomas	3	3	0		
	Epithelial neoplasms, NOS	5	3	2		
	Squamous cell neoplasms	2	1	1		
	Other	3	1	2		

Race	Asian	58	28	30	2.1873	0.9747
	American Indian or Alaska native	1	0	1		
	Black or African American	181	91	90		
	White	757	384	373		
	Unknown	85	38	47		

**Table 2:** Primer sequences for RT-PCR.

Genes	Forward primer (5′-3′)	Reverse primer (5′-3′)	Product length (bp)
GAPDH	GAAGGTGAAGGTCGGAGTC	GAAGATGGTGATGGGATTTC	227
PGK1	GTGGGGGTATTTGAATGGGAAGC	GCACAGCAAGTGGCAGTGTCTCC	124
SDC1	TTTGAAACCTCGGGGGAGAATAC	GAAACCCACCAGGCACACAGC	183

## Data Availability

The data are available at The Cancer Genome Atlas Program (TCGA, https://tcga-data.nci.nih.gov/tcga/) and Gene Expression Omnibus (GEO, https://www.ncbi.nlm.nih.gov/geo/, Accession number: GSE42568 and GSE48391).

## Supplementary Materials

Supplementary Materials Table S1: 26 hypoxia-related genes. Figure S1(a–c): the differential expression of CA9, PGK1, and SDC1 in tumor and paracancerous samples.
