# Supplementary material for: Development of an Independent Prognostic Signature Based on Three Hypoxia-Related Genes for Breast Cancer
Source: Comput Math Methods Med. 2022 Nov 3;2022:2974126. doi: 10.1155/2022/2974126 (PMC9649307; doi:10.1155/2022/2974126)
Supplement: Supplementary Materials — Table S1: 26 hypoxia-related genes. Figure S1(a–c): the differential expression of CA9, PGK1, and SDC1 in tumor and paracancerous samples. [file 2974126.f1.docx]

Table S1 26 Hypoxia-related genes

| ALDOA |
| --- |
| ANGPTL4 |
| ANLN |
| BNC1 |
| CA9 |
| CDKN3 |
| COL4A6 |
| DCBLD1 |
| ENO1 |
| FAM83B |
| FOSL1 |
| GNAI1 |
| HILPDA |
| KCTD11 |
| KRT17 |
| LDHA |
| MRGBP (C20orf20) |
| MRPS17 |
| P4HA1 |
| PGAM1 |
| PGK1 |
| SDC1 |
| SLC16A1 |
| SLC2A1 |
| TPI1 |
| VEGFA |


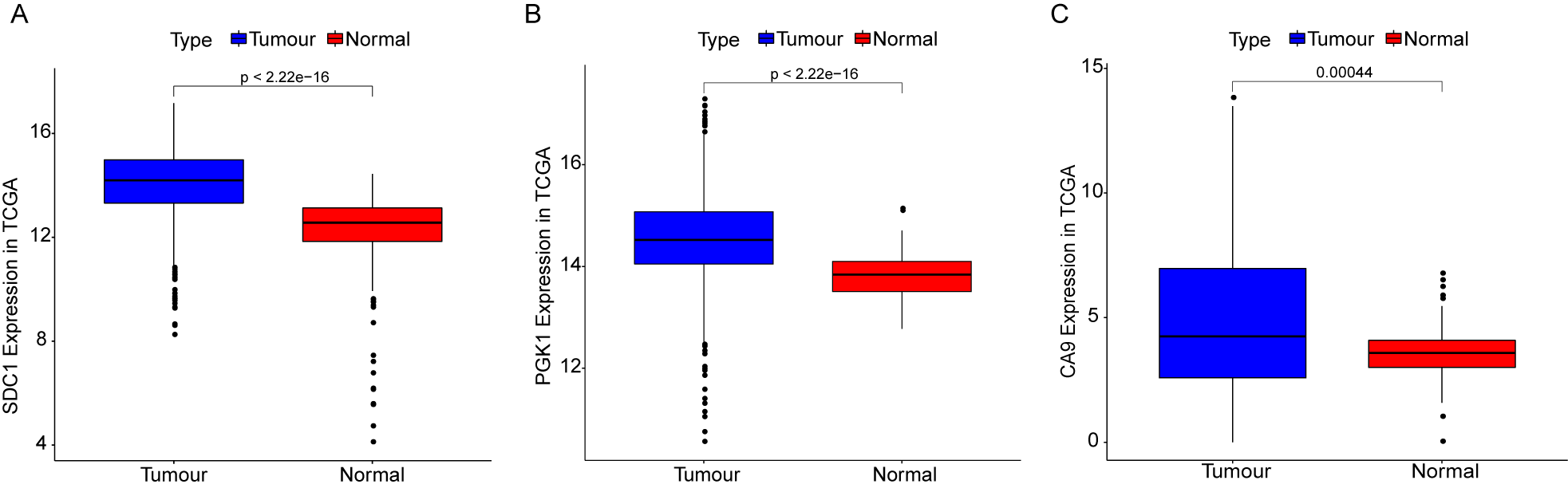


**Fig. S1 A-C** The differential expression of CA9, PGK1, and SDC1 in tumor and paracancerous samples.
